# The Dynamic Spatial Structure of Flocks

**DOI:** 10.3390/e26030234

**Published:** 2024-03-07

**Authors:** Nicholas J. Russell, Kevin R. Pilkiewicz, Michael L. Mayo

**Affiliations:** 1 Department of Mathematical Sciences, University of Delaware, Newark, DE 19716, USA; nrussell@mpipz.mpg.de; 2 U.S. Army Engineer Research and Development Center, Vicksburg, MS 39180, USA; michael.l.mayo@erdc.dren.mil

**Keywords:** collective motion, statistical physics, active soft matter

## Abstract

Studies of collective motion have heretofore been dominated by a thermodynamic perspective in which the emergent “flocked” phases are analyzed in terms of their time-averaged orientational and spatial properties. Studies that attempt to scrutinize the dynamical processes that spontaneously drive the formation of these flocks from initially random configurations are far more rare, perhaps owing to the fact that said processes occur far from the eventual long-time steady state of the system and thus lie outside the scope of traditional statistical mechanics. For systems whose dynamics are simulated numerically, the nonstationary distribution of system configurations can be sampled at different time points, and the time evolution of the average structural properties of the system can be quantified. In this paper, we employ this strategy to characterize the spatial dynamics of the standard Vicsek flocking model using two correlation functions common to condensed matter physics. We demonstrate, for modest system sizes with 800 to 2000 agents, that the self-assembly dynamics can be characterized by three distinct and disparate time scales that we associate with the corresponding physical processes of clustering (compaction), relaxing (expansion), and mixing (rearrangement). We further show that the behavior of these correlation functions can be used to reliably distinguish between phenomenologically similar models with different underlying interactions and, in some cases, even provide a direct measurement of key model parameters.

## 1. Introduction

Flocking, a phenomenon where a disordered group of self-propelled agents with a local aligning tendency spontaneously achieves some form of global self-organization, can be observed in nature across disparate biological scales ranging from birds [1,2] to bacteria [3,4], as well as in various inorganic systems, including force-driven granular media [5,6] and swarms of simple automatons [7,8]. This feature of collective motion has been intensively studied for the past several decades, but most efforts have focused on the stationary features of the fully flocked state, neglecting how those features arise over time or how they persist in fluctuating even after the flocking process has completed.

The reason for this bias is simple. Most field measurements of animal flocks consist of video recordings of a flock on the move, which, after image tracking analysis, results in a single trajectory. Due to the finite frame rate of the recording equipment, this trajectory takes the form of a discrete time series of spatial configurations, which is most suitable for estimating time-averaged, stationary properties of the flock, like its average orientational order. Because these quantities of interest tend to be defined, for any given configuration, as an average over the individual agents or pairs of agents, taking a further time average over even a short sequence of configurations is often sufficient to yield an accurate result [9,10].

For agent-based flocking models whose dynamics can be simulated numerically, it is often feasible to generate an entire ensemble of long trajectories, and this makes it possible to ask more probing questions about the dynamic evolution of the flock and the nonstationary processes that give rise to it. For example, Pilkiewicz and Eaves showed that the orientational order parameter of a simple lattice-based flocking model could be promoted to a dynamical variable by time-averaging it pointwise over an ensemble of trajectories, and they found that the saturation time scale and growth rate of this dynamic order parameter changed dramatically as the system density was increased. They associated these differences in the temporal saturation profile with disparate mechanisms of self-organization [11], but these mechanistic associations had to be made empirically since the order parameter itself contained no structural information about the system beyond its overall alignment.

Since coarse-grained continuum theories of collective motion have already demonstrated that flocking has a hydrodynamic character [12,13,14,15], the radial distribution function (RDF) traditionally used to characterize the structural properties of liquids [16] may be a more informative metric for teasing out the spatial dynamics of self-organization. This and other two-point correlation functions from condensed matter physics have been appropriated to the study of flocking in the past [17,18,19], but these other studies typically focus on static spatial correlations in the flocked steady state, whereas our objective is to consider how these spatial correlations evolve in time as the system transitions from a disordered initial state to one possessing global orientational order. Studies of this latter sort are much less common [20,21].

We focus our efforts on studying the emergence of self-organization in the ubiquitous rule-based flocking model of Tamás Vicsek and his collaborators, which has been extensively studied as a prototype of collective motion [22,23,24]. For moderate-sized systems consisting of $∼O(10^{3})$ particles, we show how the dynamic radial distribution function reveals a two-stage structural mechanism for flocking. During a short initial stage, lasting ∼100 time steps, a rapid spatial compaction process transpires, wherein locally dense, orientationally aligned clusters form and grow until global alignment is largely complete. At this point, a secondary structural relaxation process begins to take place over an order-of-magnitude longer time scale, in which the highly compact regions gradually expand outwards until a true structural steady state is reached.

Although this true steady state looks much more spatially uniform than the rapidly aligned clustered state, the RDF nonetheless exhibits a small discontinuity at the precise value of the model’s fixed interaction radius. This suggests, remarkably, that spatial correlations may be an effective tool for inferring the underlying character of the interactions driving collective motion. As a contrast, we repeat our analysis for a variant of the Vicsek model, wherein orientations are averaged over the *K*-nearest neighbors, and show that its stationary RDF is, in this case, smoothly varying across all distance scales. A similar attempt to make inferences about the underlying interactions of a Vicsek-like system using the entropy production rate [25] found comparable success, albeit at the cost of a much more complicated method of analysis than what we present here [26].

After flocking is complete, we take inspiration from studies of glassy liquids to define a novel mobility metric and utilize a variant of the van Hove correlation function [27] to study how correlations in this mobility decay temporally in the flocked steady state. This enables us to quantify yet a third time scale, this one characterizing the average time required for the flock to structurally rearrange due to the stochasticity inherent in the dynamics. We again demonstrate emergent differences in this mobility metric for the two different interaction mechanisms considered.

## 2. Modeling and Methods

As asserted, the model we study is that originally devised by Vicsek, in which *N* agents occupy a square box of sidelength *L* and choose their direction of motion at each time step by averaging over the orientations of all neighbors falling within some fixed interaction radius *R*. The agents are not infallible at calculating this average, and their tendency to error is represented through a stochastic noise contribution. The system box has periodic boundaries, and the agents all move at a constant speed $v_{0}$. For the position $r_{j}\left(t\right)$ and angular orientation $\theta_{j}\left(t\right)$ of each agent j∈{1,…,N}, these dynamical rules can be formalized mathematically as follows [28]:

(1) $$\begin{array}{cc} \theta_{j}\left(t+\Delta t\right) & =\arg\left[\sum_{j∼\ell }e^{i\theta_{\ell }\left(t\right)}\right]+\xi_{j}\left(t\right)\\ r_{j}\left(t+\Delta t\right) & =r_{j}\left(t\right)+v_{j}\left(t+\Delta t\right)\Delta t. \end{array}$$

In the above, $v_{j}\left(t\right)\equiv v_{0}\left(\cos\theta_{j}\left(t\right),\sin\theta_{j}\left(t\right)\right)$ is the velocity of each agent, and $\xi_{j}\left(t\right)$ is a discrete stochastic process whose value at each time step is uniformly chosen from the interval [−η/2,η/2], where η∈[0,2π] is the noise parameter quantifying decisional errors. If $r_{j\ell }$ is the distance between the agents located at positions $r_{j}=\left(x_{j},y_{j}\right)$ and $r_{\ell }=\left(x_{\ell },y_{\ell }\right)$, then the notation j∼ℓ denotes the set of all agents *ℓ* at time *t*, including the self contribution ℓ=j, for which $r_{j\ell }\left(t\right)\leq R$. This distance between agents must be defined with respect to the periodic boundary conditions of the system:

(2) $$\begin{array}{cc} r_{j\ell }\equiv & \left\{{\left[\min\left(|x_{j}-x_{\ell }\left|,L-\right|x_{j}-x_{\ell }|\right)\right]}^{2}\right.\\ \; & +{\left.{\left[\min\left(|y_{j}-y_{\ell }\left|,L-\right|y_{j}-y_{\ell }|\right)\right]}^{2}\right\}}^{1/2}, \end{array}$$

where min(a,b) is the standard minimum function that evaluates to *a* if a≤b and to *b* if b<a. One extracts the neighbor-averaged orientation from the complex sum in Equation (1) by applying the standard argument function from complex analysis.

For comparisons with this metric model (i.e., the interactions are based on a fixed distance), we also consider a variation in which each agent interacts with its *K*-nearest neighbors. In this topological model (i.e., the interactions involve a fixed number of connections) [29], the update rules are the same, except that now the notation j∼ℓ is interpreted as denoting the set of *K* agents *ℓ* that are closest to agent *j*. Once again, the sum will always include the self contribution j=ℓ. To ensure that a fair comparison is being made between these two variants, we choose a value of *K* such that both models have the same average number of interactions (rounded up) in their initial, uniformly selected state. We can do this by noting that, for any one agent, the average fraction of the N−1 remaining agents falling within a distance *R* of its position equals the ratio between the area of the sensory range, $\pi R^{2}$, and the total system area, $L^{2}$. The mean number of initial neighbors in the metric Vicsek model is thus equal to $\left(N-1\right)\pi R^{2}/L^{2}$, and we round this value up to the nearest integer for our value of *K* in the topological model.

When initialized from a random configuration in which the positions and orientations of each agent are chosen uniformly, both of these Vicsek systems will tend, in the absence of noise, to rapidly self-organize into a flocked state characterized by global orientational alignment. As noise is increased, this alignment is gradually degraded, and when the noise reaches a critical threshold, flocking ceases to occur. This criticality is analogous to a thermodynamic phase transition [30,31,32].

The degree of orientational order present in a given configuration of the Vicsek system is typically quantified in terms of the following order parameter:

(3) $$\sigma \equiv \frac{1}{N}\left|\sum_{j=1}^{N}e^{i\theta_{j}}\right|.$$

This parameter will be unity if all agent velocities align and will be small when the velocities are uniformly distributed. (It will only tend towards zero for a random configuration in the limit N→∞). As the noise in the dynamics is decreased, the steady-state time (or ensemble) average of this order parameter will increase from zero and saturate towards unity. It should be noted that this parameter is limited to quantifying the extent of global alignment in the system; it fails to describe any aspects of the emergent spatial structure of the flock.

### 2.1. Nonequilibrium Ensemble Averages

To quantify these structural properties and how they evolve during self-organization, we want to promote the standard radial distribution function from condensed matter physics to a dynamical variable. This promotion entails generalizing the Gibbs ensemble average of equilibrium statistical mechanics to systems that are far from equilibrium.

When simulating the Vicsek model, it is customary to initialize the system from a uniform distribution $p_{U}\left(\{r_{j}\left(0\right),\theta_{j}\left(0\right)\}\right)$ defined by the following:

(4) $$p_{U}\left(\{r_{j}\left(0\right),\theta_{j}\left(0\right)\}\right)\equiv {\left[U{\left(0,L\right)}^{2}U\left(-\pi ,\pi \right)\right]}^{N}.$$

In the above, the set $\left\{r_{j}\left(0\right),\theta_{j}\left(0\right)\right\}$ defines the initial configuration of the Vicsek system at time t=0 (the index *j* runs from 1 to *N*), and U(a,b) represents the uniform probability distribution function on the closed interval [a,b]. (Note that we have assumed, without loss of generality, that the coordinate origin lies at the lower-left corner of the system box so that both *x* and *y* coordinates for each agent fall on the interval [0,L]).

As an initial configuration evolves according to the dynamical rules of Equation (1), the configurational probability distribution function of Equation (4) will likewise evolve, with different sets of configurations becoming more or less likely to be observed with time. We will denote this time-dependent distribution as $p_{U}\left(\{r_{j}\left(t\right),\theta_{j}\left(t\right)\}\right)$. For a dynamical variable F(t) that depends upon the configuration of the system, i.e., $F\left(t\right)\equiv F\left(\{r_{j}\left(t\right),\theta_{j}\left(t\right)\}\right)$, its average value can be defined formally as follows:

(5) $$\begin{array}{cc} {\langle F\left(t\right)\rangle }_{U} & \equiv \prod_{j=1}^{N}\left[\int_{0}^{L}\int_{0}^{L}\int_{-\pi }^{\pi }dx_{j}\left(t\right)dy_{j}\left(t\right)d\theta_{j}\left(t\right)\right]\\ \; & \times F\left(\{r_{j}\left(t\right),\theta_{j}\left(t\right)\}\right)p_{U}\left(\{r_{j}\left(t\right),\theta_{j}\left(t\right)\}\right), \end{array}$$

where the *U* subscript on the angular brackets emphasizes that the average value depends upon the initial distribution of states. As t→∞ and the system reaches steady state, this average becomes equivalent to the standard Gibbs ensemble average and ceases to depend upon the initial distribution:

(6) $$\lim_{t\to \infty }{\langle F\left(t\right)\rangle }_{U}\to \langle F\rangle .$$

In the above, 〈F〉 is what we denote as the stationary value of the dynamical variable *F*.

Because the distribution $p_{U}\left(\{r_{j}\left(t\right),\theta_{j}\left(t\right)\}\right)$ is not known analytically, the average in Equation (5) must be computed by generating an ensemble of model trajectories, each of which is initialized from a configuration chosen according to the distribution in Equation (4). We can thus define ${\langle \cdot \rangle }_{U}$ as an average over an ensemble of trajectories in the same way that 〈·〉 can be defined as an average over an ensemble of equilibrium (or steady state) configurations.

A very important distinction between these two types of averages is that whereas 〈·〉 can also be computed as a time average over a single, long trajectory of the equilibrated system (assuming the system fulfills the ergodic hypothesis), the average ${\langle \cdot \rangle }_{U}$ has no such time-averaged equivalent. This means, among other things, that this latter type of average will be much more difficult to evaluate for real dynamical systems, where data are often limited to a single time series trajectory.

### 2.2. The Radial Distribution Function

Now that we have characterized how a stationary metric can be formally promoted to a dynamical variable, we define our time-dependent radial distribution function (RDF):

(7) $$g\left(r,t\right)=\frac{1}{2\pi r\rho }{\langle \frac{1}{N}\sum_{j}\sum_{\ell \neq j}\delta \left(r-|r_{j}\left(t\right)-r_{\ell }\left(t\right)|\right)\rangle }_{U}.$$

In this expression, $\rho =N/L^{2}$ is the system density, and δ(r) is the standard Dirac delta function. The interpretation of Equation (7) is straightforward: the bracketed quantity yields the average number of agents one would expect to find a distance *r* from any other agent in the Vicsek system after *t* time steps, and this quantity is divided by the value of that average in the case of a perfectly uniform spatial distribution. Given that our initialization distribution (Equation (4)) is spatially uniform by construction, g(r,t=0) will approximately equal unity. (It will exactly equal unity only in the limit N→∞).

To actually evaluate the RDF numerically, it is necessary to discretize the variable *r* into equal-sized bins of some width Δr. The optimal choice for this width depends upon the number of replicate simulations to be performed, since, while a smaller bin width leads to a better approximation of the spatially continuous RDF, a larger number of simulations become necessary to adequately sample a finer grid. For most of our calculations, we chose N=800 agents and performed 300 replicate simulations; for these values, we found that a grid width of Δr=0.04 resulted in qualitatively smooth RDF curves.

To discretize the Dirac delta function δ(r), we used the fact that it is formally the derivative of the Heaviside step function Θ(r). We can therefore rewrite Equation (7) into the form of a normalized histogram by replacing the summand with the following finite difference ratio:

(8) $$\frac{1}{\Delta r}\left[\Theta \left(r+\Delta r-|r_{j}\left(t\right)-r_{\ell }\left(t\right)|\right)-\Theta \left(r-|r_{j}\left(t\right)-r_{\ell }\left(t\right)|\right)\right].$$

As already asserted, a smaller choice for Δr makes this finite difference approximation more accurate, but at the cost of requiring a larger dataset to adequately sample the larger number of histogram bins.

### 2.3. The Mobility Correlation Function

Although the RDF for the Vicsek model approaches a stationary function, g(r), at long times, the spatial structure of the flocked state only remains perfectly static in the extreme case wherein flocking is complete and the noise parameter is zero. For a nonzero noise parameter, the individual trajectories of the agents will, over time, deviate from the mean direction of the group by varying degrees, resulting in a flock that is constantly changing, even though its average structural properties remain stationary. As Heraclitus might put it, one never steps into the same flock twice.

We would like to quantify the characteristic time scale over which these structural rearrangements occur, and thus, we define a distance metric, $\mu_{j}\left(t\right)$, that we refer to as the mobility:

(9) $$\mu_{j}\left(t\right)\equiv \left|r_{j}\left(t+T\right)-r_{j}\left(T\right)-\int_{T}^{T+t}ds\frac{1}{N}\sum_{\ell =1}^{N}v_{\ell }\left(s\right)\right|.$$

In the above, *T* is some arbitrary starting time after the system has reached the structural steady state and the sum under the integral is the agent-averaged velocity vector of the system configuration at dummy time *s*. Due to the discrete nature of the rule-based dynamics, the integral is formally a Riemann sum discretized over temporal bins of width Δt.

The mobility $\mu_{j}\left(t\right)$ quantifies how much the trajectory of agent *j* deviates from the overall average motion of the flock over the course of *t* time steps. Equivalently, $\mu_{j}\left(t\right)$ can be thought of as the magnitude of the displacement of agent *j* over *t* time steps in the reference frame of the moving flock. Because the average direction of the flock can change over time, this frame is generally noninertial. Additionally, note that for the mobility to be meaningful, we must calculate the actual distance traveled by the agent rather than the distance between its initial and final positions inside the periodic box of the model.

To quantify the time scale over which any specific steady configuration of the flock relaxes into a new, structurally similar configuration, we insert this mobility into the self component of the standard isotropic van Hove correlation function:

(10) $$G_{\mu }\left(r,t\right)=\langle \frac{1}{N}\sum_{j=1}^{N}\frac{\delta \left(r-\mu_{j}\left(t\right)\right)}{2\pi r}\rangle .$$

Note that, unlike in Equation (7), where the average was taken over an ensemble of configurations that had been evolved *t* time steps from a uniform initialization, the ensemble average here is a traditional steady-state configurational average. This means that this mobility correlation function, unlike the dynamic RDF discussed previously, can in principle be computed from a single, long, flocked trajectory—a moot point here for the Vicsek flock, but one worth noting for experimental systems for which an ensemble approach is infeasible.

Because we are now considering absolute travel distances, *r*, that can take any positive value (as opposed to periodic displacements that are constrained by the size of the simulation box), we can spatially Fourier transform this function and express it as a configurationally averaged sum of Bessel functions of the first kind:

(11) $${\tilde{G}}_{\mu }\left(k,t\right)=\langle \frac{1}{N}\sum_{j=1}^{N}J_{0}\left(k\mu_{j}\left(t\right)\right)\rangle .$$

The parameter *k* in this expression is our Fourier transform wavevector.

If the global density is sufficient for the flock to become fully aligned (σ=1) at long times, then the above function can be evaluated exactly in the case of zero noise (η=0). It can also be approximately evaluated in the case of maximal noise (η=2π). In the former case, the mobility will always be zero, so ${\tilde{G}}_{\mu }\left(k,t\right)$ will equal unity for all *t*, regardless of the choice of wavevector. In the latter case, the agents perform independent random walks and will not flock, resulting in an agent-averaged velocity that approximately equals zero at all times. For sufficiently long times, ${\tilde{G}}_{\mu }\left(k,t\right)$ will therefore reduce to the average of the Bessel function $J_{0}\left(k\right|r_{j}\left(T+t\right)-r_{j}\left(T\right)\left|\right)$ over the Gaussian distribution of a two-dimensional Wiener process. Taking this average yields ${\tilde{G}}_{\mu }\left(k,t\right)\approx \exp\left(-k^{2}Dt\right)$, where the diffusion constant for the two-dimensional Vicsek model is $D=\left(1/4\right)v_{0}^{2}\Delta t$, and the approximation becomes exact in the limit of an infinite system at long times.

## 3. Results

In the discussion that follows, all correlation functions were computed by averaging over configurations generated from 300 replicate simulations, each of which started from a different uniformly chosen initial configuration of the system and was then evolved to steady state by numerically iterating the update rules of Equation (1) for either a metric or topological interaction mechanism. In all simulations, we used a time step of Δt=1 and a fixed agent speed of $v_{0}=0.5$, whereas the values of the density, ρ, and the noise parameter, η, were varied across simulations. All model parameters are treated as dimensionless by defining them relative to an arbitrary length and time scale whose values are set to unity.

In cases where the density is varied, we always do so by changing the box length, *L*, rather than the number of agents, *N*. This is an important distinction, because the two are not equivalent. If the density is changed by scaling the box length by some factor, the interaction radius, *R*, and the distance traveled per time step, $v_{0}\Delta t$, can be scaled by the same factor to render the dynamics of the model unchanged. (This is equivalent to changing the value of the aforementioned arbitary length scale of the model to something other than unity).

In contrast, changing the density by varying the number of agents will impact the average degree of orientational order present in the initial state of the system, since it is only in the limit N→∞ that the average order parameter of a uniformly oriented state will approach zero. This difference in initial orientational bias will drive differences in the rate at which complete global alignment emerges, and there is, to our knowledge, no scaling of parameters that can counterbalance these dynamical disparities.

### 3.1. Structural Evolution

**Figure 1:**
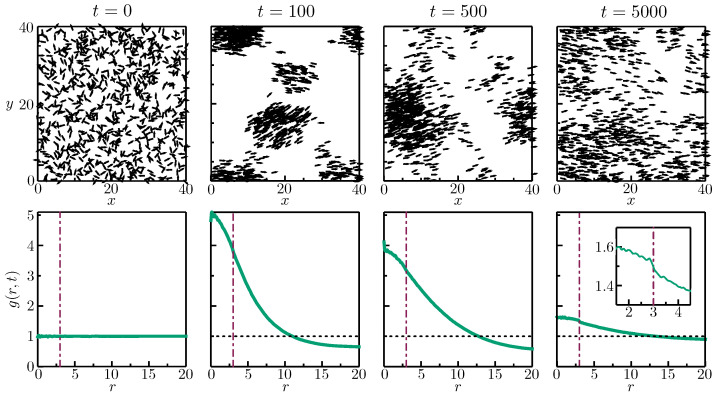
Quantifying structural evolution. Top row: Representative configurations of a Vicsek system with N=800, L=40, $v_{0}=0.5$, R=3, η=0.4, and Δt=1 for four different time points. Bottom row: Corresponding plots of the radial distribution function for the same parameter values at each of the four times. The dashed vertical line in each plot marks the value of the interaction radius, and the inset of the last plot highlights the behavior of the RDF curve near the interaction radius at steady state.

The top row of panels in Figure 1 show typical configurations for a metric Vicsek system with N=800, ρ=0.5, R=3, and η=0.4 at four different times, and the bottom row of panels plot the corresponding RDF curves for those times (averaged over 300 replicate simulations). At t=0, as expected, the system is highly disordered (σ=0.0311), and the RDF is equal to unity for all distances. In these plots, we only consider r∈[0,L/2] due to the periodic boundary conditions. Technically, the maximum distance possible between two agents is $L/\sqrt{2}$, but very few agents will sample the high end of that range.

After a mere 100 time steps, the second configuration is already predominantly aligned (σ=0.8657), and the agents have coalesced into a number of dense clumps. These large heterogeneities in the local density (giant number fluctuations) have been discussed at length elsewhere [33,34,35] and are generally viewed as a hallmark of flocking systems, much the same way that dynamic heterogeneities are considered a fundamental property of glass-forming liquids. The RDF reflects this “clumpiness” with values much larger than unity for short distances and values slightly less than unity for longer distances.

A less well-documented behavior begins after the order parameter has largely saturated. In the third portrait, after 500 time steps, the average order parameter has reached a value of σ=0.9870, but the spatial positioning of the agents has become more diffuse. The value of the RDF decreases noticeably for short distances and actually increases slightly for intermediate distances, resulting in an overall flattening of the curve. This decoarsening process continues slowly over time, with thousands of time steps elapsing before the RDF at last approaches an asymptotic limit. By t=5000, the order parameter has only marginally increased to a value of σ=0.9909, but the final pair of panels in Figure 1 show a much more uniform spatial distribution. This makes it evident that the orientational and spatial organization processes of the Vicsek system occur over disparate time scales separated by an order of magnitude.

**Figure 2:**
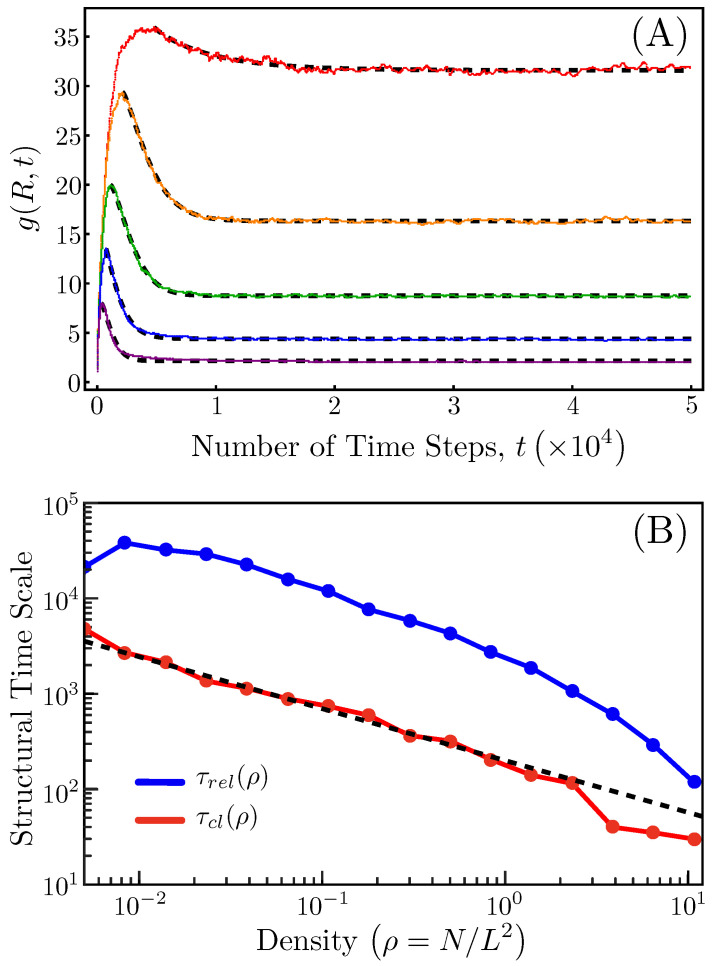
Self-assembly time scales. (**A**) Plots of g(R,t) versus time for five different densities. From top to bottom, the curves represent ρ=0.00501, 0.01394, 0.03878, 0.10791, and 0.30026. For all curves, the number of agents was kept fixed (N=2000), and the noise parameter was set at η=0.4. The dashed curves are the stretched exponential fits to each RDF curve. (**B**) The clustering time scale $\tau_{cl}$ (bottom curve) and the decoarsening time scale $\tau_{rel}$ (top curve) are plotted as a function of density for the same Vicsek conditions in (**A**) and densities ranging from 0.00501 to 10.78144. The dashed curve is a power law fit to the clustering time scale with a negative power of 0.54. Note that the curves are all plotted on a log–log scale.

To better quantify these time scales, we plot g(r=R,t), the RDF evaluated at the interaction radius (R=3), as a function of time for several different system densities (fixed N=2000) in Figure 2A. The curves all rise initially along the same roughly linear growth profile before diverting off towards different local maximum values. Because less overall compaction is required to form clusters of mutually interacting agents in higher-density systems, the maximum height of each RDF curve (a measure of the extent of compaction) and the time at which that maximum occurs both decrease with increasing density. We can define the time at which the local maximum in g(R,t) occurs as the clustering time scale $\tau_{cl}$, and we see in Figure 2B that, for densities between about $10^{-2}$ and 1, this time scale decreases roughly as a power law with a negative exponent of approximately 0.54.

After reaching a local maximum, the g(R,t) curves slowly decay towards their long-time steady values. Again, because denser systems do not coarsen as drastically in the first place, their subsequent decoarsening is faster and more complete. Consequently, the long-time RDF value, i.e., the degree of nonuniformity present at steady state, and the time it takes for this value to be reached both decrease with increasing density. This decay can be well fit by a shifted stretched exponential function of the following form:

(12) $$\alpha \left\{\exp\left[-{\left(\frac{t-\tau_{cl}}{\tau }\right)}^{\beta }\right]-1\right\}+g\left(R,\tau_{cl}\right),$$

where α, β, and τ are the fitted parameters. The relaxation time for a stretched exponential is typically reported as the area underneath it, so shifting the long-time asymptote of the function in expression (12) down to zero (which does not impact the shape of the curve) and integrating the function over the interval $\left[\tau_{cl},\infty \right]$, one obtains a relaxation time scale $\tau_{rel}$ for the decoarsening of the Vicsek flock:

(13) $$\tau_{rel}=\frac{\alpha \tau }{\beta }\Gamma \left(\frac{1}{\beta }\right),$$

where Γ(z) is the standard gamma function. Note that when β=1, $\tau_{rel}$ reduces to ατ, which is the relaxation time scale for an ordinary exponential function. The time scale $\tau_{rel}$ is also plotted versus system density in Figure 2B, and we see that it is roughly an order of magnitude larger than $\tau_{cl}$, though this difference diminishes for very dense systems.

Clearly, the driving force behind this spatial decoarsening must be the stochastic noise, as without noise, the agents would translate through space in lockstep once aligned. As one can see from the curves in Figure 2A, this gradual smoothing of the local density does not continue until the spatial uniformity of the initial state is recovered, and one can even see from the bottom-right panel of Figure 1 that the long-time steady state of the system remains visibly clumpy over shorter distance scales. This persistent nonuniformity can be understood as resulting from a balance between the entropic (noisy) and energetic (aligning) contributions to the dynamics, even though the Vicsek system formally has no configurational energy. For very large system sizes ($L∼O(10^{2})$, same density), this balance of forces results in system-spanning bands in addition to persistent clumps, which have been reported elsewhere [36]. We continue to restrict our attention to modest-sized flocks, however, as our interests ultimately lie towards the application of these analytical methods to real, finite animal flocks and not in probing the thermodynamic limit of agent-based models.

**Figure 3:**
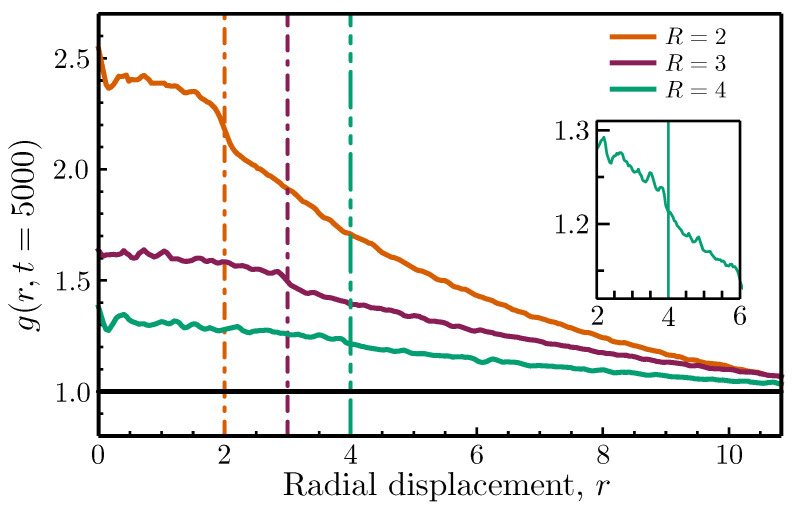
The steady-state radial distribution function for three different interaction radii. The three curves correspond to plots of the Vicsek model RDF computed at steady state for interaction radii (from top to bottom) R=2, 3, and 4. All other parameters are the same as those used in Figure 1. Vertical lines mark each interaction radius value for clarity, and an inset shows a zoomed-in view of the bottom curve to emphasize its behavior near *R*.

If the above interpretation is correct, then we would expect the interaction radius to play a significant role in controlling the degree of nonuniformity observed in the long-term spatial structure of the Vicsek flock. In the extreme case of global-ranged interactions, the flock will align immediately, predominantly leading to a preservation of the initial uniform structure; for shorter interaction distances, the agents must first move into closer proximity before interacting, resulting in the formation of smaller, locally aligned clusters that must gradually coalesce and merge before a significant degree of global orientational order can be achieved. This suggests that the steady state of the system should be less uniform for shorter interaction radii, which is precisely what we observe in Figure 3, wherein the RDF is plotted at t=5000 for three different values of *R*.

What is especially fascinating is that each of the curves in Figure 3 exhibits an almost discontinuous drop coincident with the value of the interaction radius. While this feature is most pronounced for the smallest value of *R*, the insets in both Figure 3 and the bottom-right panel of Figure 1 demonstrate that this drop is present for the two larger interaction radii as well, albeit suppressed due to the greater uniformity of the spatial structure. This feature indicates that the specific value of the interaction radius leaves an indelible mark on the long-time spatial structure of the flock that is not obvious to the naked eye and which cannot be captured by the standard orientational order parameter. Importantly, this means that, given trajectory data from a metric Vicsek system of unspecified system parameters, a plot of the stationary RDF could be used to extract the unknown value of the interaction radius.

**Figure 4:**
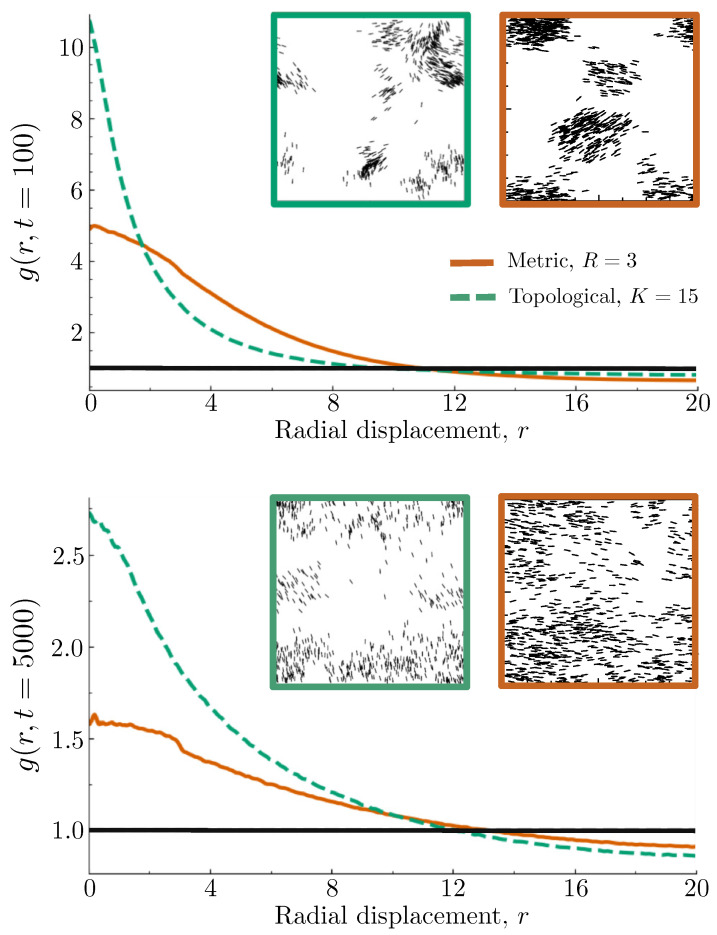
Radial distribution function for metric and topological Vicsek variants. **Top** panel: The RDF for the metric (solid curve, R=3) and topological (dashed curve, K=15) Vicsek models at time t=100. All other parameters are the same as those used in Figure 1 for both models. The left inset shows a typical configuration of the topological system at t=100, and the right inset shows the same for the metric model. **Bottom** panel: The same plot (and insets) for t=5000. Note that only the metric curve develops a discontinuous drop at r=R.

However, what if the trajectory data in question were generated not by a metric Vicsek model, but by a topological one? The small discontinuity at the value of the interaction radius can be difficult to distinguish from statistical noise in cases where the time series is not long enough for robust configurational averaging, so that feature alone may not be sufficient for reliably determining the nature of the interaction mechanism responsible for the observed self-organization. Indeed, one might expect there to be little difference in the structural dynamics of these two Vicsek model variants, since a topological interaction rule will not introduce many long-ranged interactions once the system has compacted into dense, localized clusters; but Figure 4 shows that, although the spatial dynamics of both models exhibit an initial compaction stage followed by a slower relaxation stage, the spatial structures produced by these dynamics remain quantifiably distinct.

In the top panel of Figure 4, the RDF curves for a metric Vicsek model with R=3 and a topological Vicsek model with K=15 are plotted after 100 time steps have elapsed. Recall that we chose the corresponding values of *R* and *K* so that agents in both models initialized with the same average number of interactions. We see that the topological model RDF is much larger than its metric model counterpart for small distances, but smaller for intermediate distances. The representative configurations shown as insets to the plot make it clear why this is the case: while an initial compaction occurs in both systems, the clusters formed through the topological interaction mechanism are smaller and denser than those formed by the standard metric mechanism. Although a structural relaxation follows in both systems, the bottom panel illustrates that, at steady state (t=5000), the topological system is still locally more dense and compact than the metric system. As expected, the discontinuous drop in the RDF that emerges at r=R for the metric model does *not* emerge in the topological model. Even in cases where a lack of data makes robustly determining whether such a discontinuity exists or not, the metric and topological interaction mechanisms clearly produce RDF curves with disparate profile shapes, with the former being much flatter and more slowly varying over small to intermediate distance scales.

Interestingly, the reason the topological Vicsek model evolves into a more compacted spatial structure than its metric model counterpart is not a consequence of a longer effective interaction radius, but rather a shorter one. When two clusters collide in the metric model, agents near the collisional front of each cluster begin to interact with each other while continuing to interact with the rest of the agents in their own respective clusters. This leads to both clusters slowly aligning towards the same average direction and merging into a single, large cluster.

When the same collision occurs in the topological model, the fixed number of interactions requires the two clusters to get much closer together before agents in different clusters begin to interact. Furthermore, agents near the front of the clusters will interact largely with agents in the other cluster, while those towards the back of each cluster will continue to interact exclusively with each other. Consequently, the agents nearest the collisional front will rapidly turn to the new averaged direction of the two clusters while the remaining agents keep moving in their original directions until they too collide. The end result is a sort of throttling process that results in denser, narrower groupings than those produced by the metric model, such as what happens when a large crowd escapes through a narrow door.

### 3.2. Steady-State Dynamics

**Figure 5:**
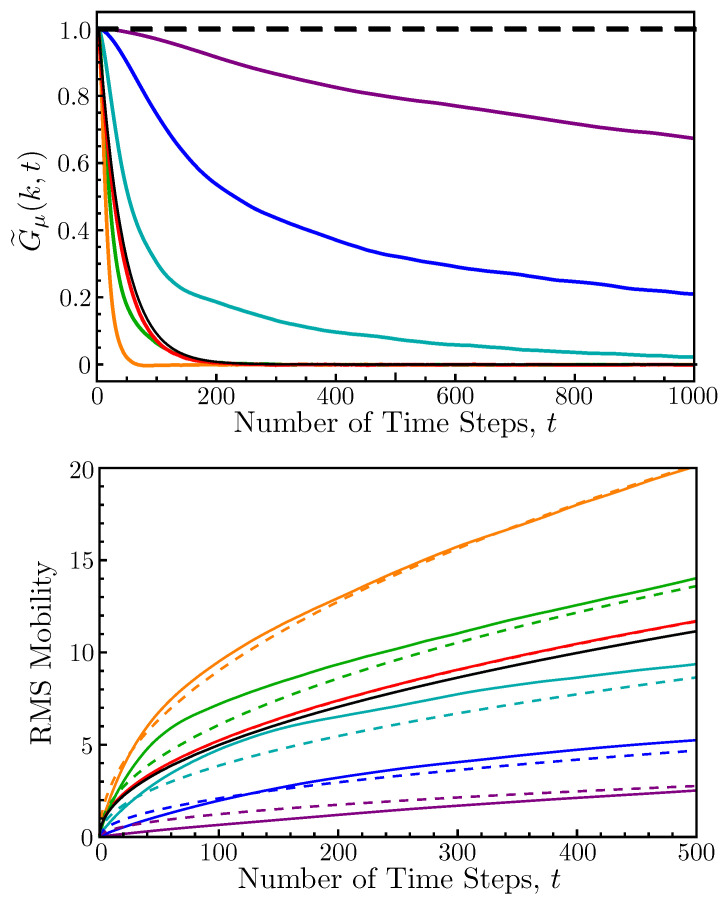
The mobility correlation function and RMS mobility. **Top** panel: Plots of the mobility correlation function ${\tilde{G}}_{\mu }\left(k,t\right)$ for k=2π/10 and eight different values of the noise parameter η. From top to bottom, the curves correspond to η=0, 0.1, 0.3, 1.0, 2π, 5.5, 2.5, and 3.5. All other parameters of the metric Vicsek system are the same as those used in Figure 1. Note that for intermediate values of the noise, the curves decay more rapidly than in the diffusive limit (η=2π). **Bottom** panel: The root mean squared mobility plotted vs. time for the same eight noise values (now arranged from bottom to top). The η=0 curve coincides with the abscissa. The dashed curves of matching color fit the long-time power law growth of the RMS mobility to a standard diffusion rate law ${\left(4D_{eff}t\right)}^{1/2}$.

The top panel of Figure 5 plots the mobility correlation function ${\tilde{G}}_{\mu }\left(k,t\right)$ versus time for k=2π/10, a fixed density of ρ=0.5, and different amounts of noise in the metric Vicsek model (R=3). This correlation function decays more rapidly as *k* increases, so we chose the value k=2π/10 simply because it allowed the curves for the full range of sampled noise parameter values to fit clearly on a single plot. As η increases, one might expect the structural reorganization time scale of the flock to monotonically interpolate between the two limiting values of infinity and ${\left(k^{2}D\right)}^{-1}$ (for η=0 and 2π, respectively); but for intermediate noise values, the mobility correlations actually decay more rapidly than in the diffusive limit. It is only after a certain critical noise value that structural rearrangements begin to slow down again, asymptotically approaching the diffusive curve from below (rather than above) as η→2π.

In the moving frame of the flock, the stochastic process driving these structural reorganizations is approximately diffusive for any value of the noise parameter, a fact that can be most easily demonstrated by examining how the root mean squared (RMS) mobility scales with time. The bottom panel of Figure 5 plots the RMS mobility versus time for the same values of η used in the top panel (solid curves) and fits each curve to a power law of the form ${\left(4D_{eff}t\right)}^{1/2}$ (dashed curves). The parameter $D_{eff}$ is an effective diffusion constant that depends upon the noise. For intermediate values of the noise, these diffusive fits are especially bad at short times; but for all values of η, the curves capture the correct power law scaling at long times. This suggests that, at least to a leading order, we can characterize the mixing time scale of the steady Vicsek flock as follows:

(14) $$\tau_{mix}\approx {\left(k^{2}D_{eff}\right)}^{-1}.$$

The dependence of this time scale on the wavevector indicates that mixing occurs at different rates over different length scales, which is a sensible conclusion. The rearrangement of the entire flock should take longer than the rearrangement of a small, localized cluster of agents.

The quasi-diffusive behavior of the Vicsek agents in the moving frame of the flock is quite similar to the laboratory-frame behavior of agents in run-and-tumble models—essentially random walkers that only change their direction (tumble) occasionally and move ballistically (run) the rest of the time [37]. The mean squared displacement of run-and-tumble agents is diffusive for long times, but superdiffusive at short times, which is consistent with what we observe for the mean squared mobility in the Vicsek system.

**Figure 6:**
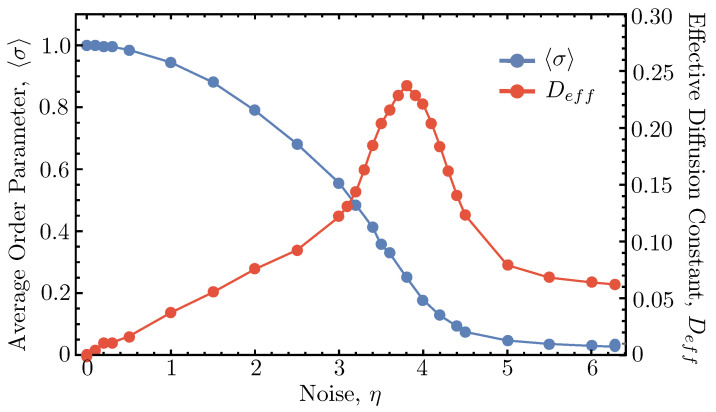
Comparison of 〈σ〉 and $D_{eff}$ vs. noise. The average order parameter of the metric Vicsek model at steady state is plotted vs. noise (η) in blue for the same simulation parameters used in Figure 1. The effective diffusion constant vs. noise curve is overlaid in red on the same plot with a different vertical scale to emphasize that the maximal value of $D_{eff}$ and the inflection point of the average order parameter curve roughly coincide at the same value of η.

As noise is increased, localized clusters of agents become more likely to make ballistic “runs” in a direction different from the overall mean direction of the flock, which leads to faster mixing. Eventually, however, a critical noise value is reached above which stochasticity begins to win out over the aligning interaction of the dynamics, resulting in the agents behaving increasingly like standard diffusive walkers. This nonmonotonic behavior of the effective diffusion constant as a function of the noise is plotted in Figure 6. The average order parameter versus noise curve is overlaid (with a different vertical axis) to demonstrate that the maximal value of $D_{eff}$ (fastest mixing time scale) corresponds roughly with where the average order parameter has its inflection point (η≈3.5). This inflection point denotes where the extent of flocking is maximally susceptible to small changes in the amount of noise, which corresponds to a state of high dynamic instability.

**Figure 7:**
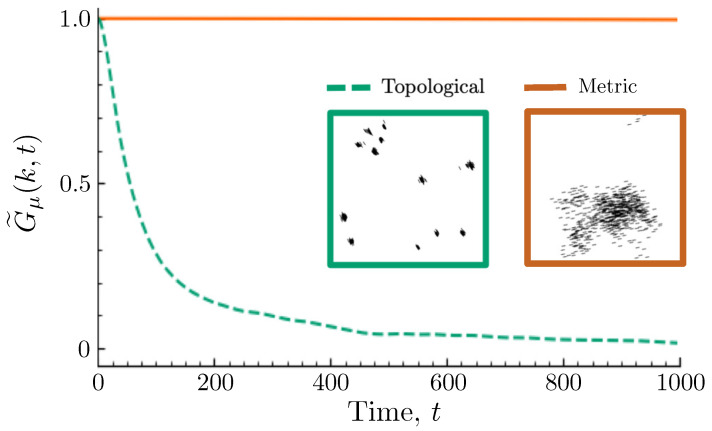
Comparison of zero-noise mobility correlations. Plots of the mobility correlation function ${\tilde{G}}_{\mu }\left(k,t\right)$ for k=2π/10 and η=0 for the metric and topological models at R=3 and K=15, respectively. All other parameters of the models are the same as those used in Figure 1. Although both models flock robustly under these conditions, the spatial structure of the topological Vicsek flock gradually changes over time, whereas the metric model flock retains a rigid structure. The origin of this difference can be understood from the representative configurations of each model, shown as insets (the topological configuration is on the left, the metric on the right), which illustrate the vastly different spatial distributions of the two models.

Interestingly, while our expectation that there should be zero mobility in the absence of noise is borne out for flocked configurations in the metric Vicsek model, this assumption does not hold in general. If we once again replace the metric aligning interaction with a topological one (K=15), we see in Figure 7 that, although both zero-noise systems achieve near-complete saturation of the average order parameter at long times (for the specific model parameters considered, 〈σ〉=0.99997 in the metric model and 0.99345 in the topological model), the mobility correlation function ${\tilde{G}}_{\mu }\left(k,t\right)$ still decays over time for the topological flock.

Representative configurations of the two steady states (shown as insets in Figure 7) reveal the origin of this disparity to be the more severe structural compaction of the topological model. This compaction results in numerous small clusters aligned in slightly different directions that cannot collide and merge due to how sparsely they are distributed about the area of the simulation box. Even though the differences in their directions of propagation are small, they are enough to cause the relative positions of different clusters to change over time, resulting in a gradual structural relaxation of the system. In the metric model, on the other hand, initially formed clusters are less compacted and are consequently able to collide and merge until a single fully aligned cluster is achieved. (Note that the small grouping of agents at the top of the box in the depicted configuration is connected to the rest of the agents due to the periodic boundary conditions).

## 4. Discussion

In this paper, we have demonstrated how an ensemble of numerically simulated model trajectories can be used in conjunction with dynamic, two-point correlation functions to probe the mechanistic details of flocking. For moderate system sizes of the standard Vicsek flocking model ($N∼O(10^{3})$), our approach revealed three key time scales characterizing the structural dynamics of the model. The first, $\tau_{cl}$, describes the compaction of the initially uniform system into locally aligned clusters that collide and merge until a global orientational order is achieved. Though this mechanism rapidly generates orientational order across the system, it results in a “clumpy” spatial structure that is highly nonuniform.

Even though the average order parameter is very close to unity at this point, the spatial structure of the flock continues to undergo a secondary relaxation process described by a much longer time scale, $\tau_{rel}$. In this process, noise drives high-density regions of the flock to relax and expand, gradually smoothing out the nonuniformity of the system until a true steady state is achieved. Some nonuniformity in the spatial structure does persist, however, and we have shown, for the standard Vicsek model with a metric interaction mechanism, that the precise character of this structure encodes the fixed value of the agent interaction radius.

Even after a steady state has been reached, the Vicsek flock continues to evolve over time. Though its average spatial properties are locked in, so to speak, the positions of individual agents in relation to one another remain in flux. Deviations from the mean direction of collective motion gradually drive rearrangements in the flock from one structurally similar configuration to another, and we have extracted this mixing time scale, $\tau_{mix}$, from the decay rate of our Fourier-transformed mobility correlation function. The dependence of this time scale on the magnitude of the Fourier wavevector can be used to quantitatively compare how mixing times vary over different length scales.

It is worth emphasizing that our efforts here are not meant to be interpreted as an exhaustive cataloging of the possible structural dynamics observable in the Vicsek system. Finite size effects play an important role in this model, especially far from the hydrodynamic limit studied by Toner and Tu [12], and there are certainly structural features, such as the emergence of dense, system-spanning bands [36], that only emerge in Vicsek systems with much larger numbers of agents (even at densities comparable to those we have considered).

While our correlation function analysis could certainly, in principle, be leveraged to better understand the structural dynamics of these larger systems, the computational cost would be significant. Averaging over an ensemble of configurations with a much larger number of agents is perfectly tractable, but pointwise averaging over an ensemble of *trajectories* is much more intensive—especially since flocking tends to occur more gradually in larger systems, therefore necessitating longer trajectories to fully characterize the dynamics of structural self-organization.

The phenomenon of collective motion is not limited in nature to massive starling flocks or dense clouds of midges; it is observable even among modestly sized groups of social organisms that lie far from any sort of thermodynamic or continuum limit, and we have demonstrated that our correlation function methods, despite having their origin in the statistical thermodynamics of equilibrium condensed matter systems, can still be successfully applied at this reduced scale. It remains to be seen how compactification/relaxation time scales change when alignment competes with other motile phases and structural factors. For example, rafting motile fire ants locally align in response to weak pairwise interactions [38], yet the raft bulk contracts due to a net inward flow of ants. Here, individual ants transition between states in which they either bind together into a raft floor or freely move over the top of it. These two states are intimately linked, and both contribute to the dynamic stability of the rafting structure.

As our comparative study of the metric and topological Vicsek models hopefully demonstrated, a major advantage of employing these correlation functions is that they can sometimes reveal important details about the underlying dynamical interactions that drive collective motion—details that are not generally known a priori when studying the flocking of real living organisms. Computing these correlation functions from organism trajectories measured in the field may thus provide a means for extracting useful information about the cognitive decision making and social forces that drive observed group behaviors [39].

Although acquiring data for a large number of replicate trajectories may be prohibitively costly in the field or laboratory, many of the structural differences we observed between the metric and topological Vicsek models came from steady-state calculations that can equivalently be computed from a single, long flocked trajectory. Thus, for example, if movement decisions in an animal group principally depend on sensory inputs with a finite range, we might expect the steady-state RDF to reveal this through a fairly flat functional profile over small to intermediate distance scales that undergoes a roughly discontinuous drop at the value of this sensory radius. Similarly, if a highly flocked system (low noise) is found to exhibit a rapidly decaying mobility correlation function, it is likely that individual organisms are only capable of paying attention to a fixed number of their fellows due to cognitive limitations, even if a much larger number of confederates fall within the range of their senses.

## Data Availability

The original code used to perform all numerical simulations and statistical computations may be found at https://github.com/ricknussell31/Dissertation_Code (accessed on 4 May 2021).
